# Incidence of Musculoskeletal Injuries in Natural and Artificial Turfs during the 2024 First Division of Brazilian Football Championship: A Retrospective Study

**DOI:** 10.1055/s-0046-1822977

**Published:** 2026-07-28

**Authors:** Matheus Martins Godoy, Jéssica Chávare, Pedro Lucas Leal Chaves, João Pedro Ferreira de Almeida, Matheus Alves de Carvalho Freitas, Gabriel Moraes de Oliveira, Flávia Costa Oliveira Magalhães, Israel Teoldo da Costa

**Affiliations:** 1 Centro Universitário de Patos de Minas (UNIPAM), Patos de Minas, MG, Brazil; 2 Universidade Professor Edson Antônio Velano (UNIFENAS), Belo Horizonte, MG, Brazil; 3 Centro Universitário de Belo Horizonte (UNIBH), Belo Horizonte, MG, Brazil; 4 Universidade Federal do Recôncavo da Bahia (UFRB), Santo Antônio de Jesus, BA, Brazil; 5 Massachusetts General Hospital, Harvard Medical School, Boston, MA, United States; 6 Escola Paulista de Medicina, Universidade Federal de São Paulo, São Paulo, SP, Brazil; 7 Universidade Federal de Minas Gerais (UFMG), Belo Horizonte, MG, Brazil; 8 Center of Research and Studies in Soccer (Núcleo de Pesquisa e Estudos em Futebol, NUPEF), Department of Physical Education, Universiidade Federal de Viçosa (UFV), Viçosa, MG, Brazil

**Keywords:** ankle, knee, musculoskeletal system/injuries, soccer, sports, trauma, esportes, futebol, joelho, sistema musculoesquelético/lesões, tornozelo, trauma

## Abstract

**Objective:**

To describe and compare matches played on artificial and natural turfs during the 2024 Campeonato Brasileiro Série A (the championship of the highest level of the professional association football league in Brazil), focusing specifically on the observed incidence and distribution of musculoskeletal injuries.

**Methods:**

In the current retrospective observational study, we analyzed all 380 matches of the 2024 season. Data regarding turf type, match characteristics, and injuries were obtained exclusively from publicly available secondary sources, including the official website of Confederação Brasileira de Futebol (CBF; https://www.cbf.com.br ) and major national media outlets. Cases with conflicting information were excluded. Exposure during the match was estimated assuming 22 players and 90 minutes, with incidence expressed as injuries per 1 thousand player-hours. Sub-analyses excluded previous reinjuries and direct trauma cases.

**Results:**

A total of 88 injuries was recorded, showing 6.6 injuries per 1 thousand player-hours on natural turf versus 9.5 injuries per 1 thousand player-hours on artificial turf. Hamstring strains and ankle sprains were the most frequently observed types. Regarding the turfs, thermoplastic elastomer (TPE) turf presented the highest rates among the artificial ones, while perennial ryegrass presented the highest rates among the natural ones. Injury numbers varied by context, with considerable rates observed regarding artificial turf during afternoon or night matches and at lower temperatures. Surgical treatments were frequently observed in association with natural turf, whereas recovery times differed minimally. Notably, one team whose stadium used artificial turf home team and one team whose stadium used natural turf recorded identical injury totals.

**Conclusion:**

Differences in injury occurrence between turf types were minimal. Given the retrospective design, secondary data reliance, underreporting potential, and limited control of confounders, the findings represent observational associations. Future prospective studies using standardized surveillance are warranted.

## Resumo

**Objetivo:**

Descrever e comparar partidas disputadas em gramado sintético e gramado natural na Série A do Campeonato Brasileiro de Futebol, com foco específico na incidência e na distribuição observadas de lesões musculoesqueléticas.

**Métodos:**

Neste estudo observacional e retrospectivo, foram analisados todos os 380 jogos da temporada de 2024. Os dados sobre o tipo de gramado, as características das partidas e as lesões foram obtidos exclusivamente de fontes secundárias acessíveis ao público, como o *site* oficial da Confederação Brasileira de Futebol (CBF; https://www.cbf.com.br ) e os principais meios de comunicação nacionais. Foram excluídos casos com informações conflitantes. A exposição durante as partidas foi estimada, presumindo 22 jogadores e 90 minutos, com a incidência expressa como lesões por mil horas-jogador. Subanálises excluíram relesões anteriores e casos de trauma direto.

**Resultados:**

Um total de 88 lesões foi registrado, o que indica 6,6 por mil horas-jogador em gramado natural, em comparação com 9,5 por mil horas-jogador em gramado sintético. As lesões nos isquiotibiais e as entorses de tornozelo foram os tipos mais frequentemente observados. Quanto aos gramados, o termoplástico apresentou as maiores taxas entre os sintéticos, ao passo que o azevém perene apresentou as maiores taxas entre os naturais. O número de lesões variou conforme o contexto, com taxas notáveis observadas em gramado sintético durante as partidas da tarde ou da noite e em temperaturas mais baixas. Os tratamentos cirúrgicos foram observados frequentemente com relação ao gramado natural, ao passo que os tempos de recuperação diferiram minimamente. Notavelmente, uma equipe cujo estádio era de gramado sintético e outra cujo estádio era de gramado natural registraram totais de lesões iguais.

**Conclusão:**

As diferenças na ocorrência de lesões entre os tipos de gramado foram mínimas. Considerando-se o desenho retrospectivo, a confiança nos dados secundários, o potencial de subnotificação e o controle limitado de fatores de confusão, os resultados representam associações observacionais. Estudos prospectivos com vigilância padronizada são necessários.

## Introduction

The development of artificial turf, widely used in global football, began in the late 1990s, and in 2004 the International Association Football Federation (Fédération Internationale de Football Association, FIFA, in French) authorized the use of third-generation artificial turf in official matches. 1 2 3 Despite its worldwide adoption, concerns persist regarding its potential to cause serious injuries in professional players. Musculoskeletal injuries are believed to result from factors such as turf type and properties, 2 4 5 6 7 footwear characteristics, 8 9 10 overuse, and impact trauma, 11 12 13 all considered major contributors.

Recent systematic reviews and meta-analyses have sought to clarify the relationship between playing surface and injury risk. Gould et al. 6 reported overall similar lower-limb injury rates in the comparison between artificial and natural turf, although ankle injuries appeared more frequently on artificial surfaces. Likewise, Kuitunen et al. 8 demonstrated comparable injury incidence in the comparison between surfaces, with only modest increases in specific injury types on artificial turf. Meyers 10 found no clinically-relevant differences in overall injury severity in the comparison between artificial and natural turf, despite variations in injury mechanisms. These findings suggest that, although artificial turf may influence certain injury patterns, its overall risk profile remains comparable to that of natural turf.

In Brazilian football, Campeonato Brasileiro Série A (the highest level of the professional association football league in Brazil) is among the most competitive and prestigious leagues, featuring top clubs and elite athletes. The playing surface—natural or artificial—strongly influences performance and injury risk. 14 15 16 However, differences in climate, match congestion, and turf maintenance, as well as surface heterogeneity, may limit the direct applicability of international findings to the Brazilian professional context. Although injuries are frequent, evidence suggests turf type affects incidence, with artificial surfaces linked to higher risk of traumatic injury. 4 17 18

The 2024 season of Campeonato Brasileiro Série A provided an ideal context to analyze musculoskeletal injuries across different surfaces. The alternation between turf types, dense match schedule, and intense play may increase injury risk, emphasizing the importance of assessing field conditions and athlete health. 19 20 Therefore, the present study aimed to analyze the incidence and characteristics of musculoskeletal injuries occurring on artificial and natural turf during the 2024 season of Campeonato Brasileiro Série A. The research hypothesis was that matches played on artificial turf would be associated with a higher incidence of musculoskeletal injuries, particularly involving the lower limbs, compared with those played on natural turf.

## Methods

The current retrospective observational study analyzed all 380 matches of the 2024 Campeonato Brasileiro Série A, played between April and December. Information regarding the type of turf used in each stadium was obtained from the official websites of the companies responsible for pitch installation and maintenance.

The official website of the Brazilian Football Confederation (CBF) ( https://www.cbf.com.br ) served as the primary web-based data source, providing match reports, injury notifications, schedules, and related information. These data were complemented by publicly-available records from major sports media outlets (Globo Esporte: https://ge.globo.com ; ESPN: www.espn.com.br ; and TNT Sports: www.tntsports.com.br ) to obtain additional details on injury characteristics. Data extraction was performed using more than one source whenever possible, and cases with inconsistent or conflicting information were excluded.

Because the study relied exclusively on secondary, publicly-available data, injury reporting depended on information released by clubs and media sources. Consequently, incomplete reporting, underreporting—particularly of minor injuries—and diagnostic misclassification could not be ruled out and were considered inherent limitations of the study design. We did not obtain access to official medical records or standardized injury surveillance systems.

Data collection covered all 380 matches through the 38th round and included the following variables: occurrence of injury, traumatic or non-traumatic mechanism, match withdrawal, exposure to artificial turf in the previous match, number of matches since last artificial turf exposure, reported injury history in the same anatomical region, estimated absence duration, and type of treatment (conservative or surgical). Musculoskeletal injuries were operationally defined as injuries affecting muscles, tendons, ligaments, joints, or bones that were reported during or immediately after a match and resulted in medical care or match absence. This operational definition was adapted from established injury-surveillance frameworks proposed by FIFA, the Union of European Football Associations (UEFA), and the International Olympic Committee (IOC), and we acknowledge that the lack of direct clinical assessment and standardized reporting prevented full adherence to international consensus criteria. 21 22

Match distribution according to turf type was as follows: natural turf—172 matches on Celebration Bermuda (Sod Solutions, Inc), 29 on TifGrand Bermuda (Atlanta Sod Company), 43 on Tifway 419 Bermuda, 22 on *esmeralda natural* grass, and 59 on perennial ryegrass; artificial turf—19 matches on Coolplay (SYNLawn Australia/APT), 19 on Geofill (Italgreen S.p.A.), and 16 on thermoplastic elastomer (TPE).

Additional variables included match time, environment temperature at kickoff, injury mechanism, presence of direct trauma, reported footwear adequacy, number of matches played without artificial turf exposure, and treatment approach. Exposure during the match was estimated assuming 22 players per match and a 90-minute duration, corresponding to 33 player-hours per match. Injury incidence was expressed as injuries per 1 thousand player-hours to account for exposure imbalance. In total, 49 injuries were excluded from recovery-time analyses due to unavailable return-to-play information.

Sub-analyses were conducted excluding injuries with a reported history in the same anatomical region and those clearly associated with direct trauma, aiming to describe injury incidence under non-traumatic and first-event conditions.

### Statistical Analysis

A statistical analysis was conducted to estimate the incidence and prevalence associated with factors that may influence the occurrence of injuries and those related to the type of turf present in the analyzed matches. In this context, statistical analyses were performed to assess the association between the type of playing surface and the incidence of injuries in official matches. Descriptive statistics were used to calculate absolute and relative frequencies. Injury incidence was calculated as the number of injuries per 1 thousand player-hours to account for the unequal number of matches and exposure time between natural and artificial turf. Additionally, the Mann-Whitney U test was applied to assess the association between categorical and scalar (non-parametric, as confirmed by the Kolmogorov-Smirnov test) variables of interest, adopting a statistical significance level of *p* < 0.05.

Following the same significance level, the Chi-squared (χ ^2^ ) test was used to assess the association between categorical variables of interest. Furthermore, odds ratios (ORs) and prevalence ratios (PRs), along with their respective 95%CIs, were calculated to analyze the investigated variables with the occurrence of injuries and the type of treatment adopted in response to potential risk factors. Incidence rate ratios (IRRs) and corresponding 95%CIs were estimated using Poisson regression models with log (player-hours) included as an offset variable.

Logistic regression was not performed because the available data were aggregated at the match level and did not include individual player-level exposure or binary outcome data required for valid probability modeling. Additionally, no multivariate adjustment was performed for potential confounding factors due to a lack of consistent and reliable information on these variables in publicly-available data sources.

As the current study included all official matches of the season under investigation, no a-priori sample size or statistical power calculation was performed. Effect estimates were therefore interpreted based on precision and confidence intervals. All analyses were performed using the IBM SPSS Statistics for Windows (IBM Corp.) software, version 23.0.

## Results

The present study analyzed the 2024 Campeonato Brasileiro Série A, which included 20 teams playing 38 matches each (380 total) from April 13 to December 8, 2024. Games were held in 20 stadiums—3 with artificial turf and 17 with natural grass. Consequently, 3 teams played home matches on artificial turf and 17, on natural grass. The artificial turfs included TPE, Coolplay, and Geofill. Natural turfs included Celebration Bermuda, TifGrand Bermuda, Tifway 419 Bermuda, *esmeralda natural* , and perennial ryegrass ( Table 1 ). Exposure during the matches was estimated assuming 22 players per match and a standard match duration of 90 minutes, corresponding to 33 player-hours per match.

**Table 1:** Stadiums with your Respective Grasses. Table created using Microsoft Word (Microsoft Corp., Redmond, WA, USA)

Stadium	Artificial	Type
**Artificial Stadium 1**	**YES**	**Geofill**
Natural Stadium 1	NO	Bermuda Celebration
Natural Stadium 2	NO	Bermuda Celebration
Natural Stadium 3	NO	Bermuda Celebration
**Artificial Stadium 2**	**YES**	**Cool Play**
Natural Stadium 4	NO	Bermuda Celebration
Natural Stadium 5	NO	Perennial Ryegrass
Natural Stadium 6	NO	Esmeralda Natural
Natural Stadium 7	NO	Bermuda Celebration
Natural Stadium 8	NO	Bermuda Tifgrand
Natural Stadium 9	NO	Bermuda Celebration
Natural Stadium 10	NO	Bermuda Celebration
Natural Stadium 11	NO	Bermuda Celebration
Natural Stadium 12	NO	Bermuda Tifgrand
Natural Stadium 13	NO	Perennial Ryegrass
Natural Stadium 14	NO	Perennial Ryegrass
**Artificial Stadium 3**	**YES**	**Thermoplastic (TPE)**
Natural Stadium 15	NO	Bermuda Celebration
Natural Stadium 16	NO	Bermuda Tifway 419
Natural Stadium 17	NO	Bermuda Tifway 419

### Statistical Analysis

Injury incidence by grass type was calculated using the ratio between recorded injuries and total matches played per surface. Relative frequencies, such as matches on Coolplay turf, were used to evaluate injury probability per condition. A positive yet subtle association emerged between turf type and injury number (Mann-Whitney U = 7336; Z = -2.036; *p* = 0.042), indicating a higher observed number of injuries per match on synthetic turf, without sufficient statistical evidence to establish causality. Injury history was not associated with the number of injuries per match (Mann-Whitney U = 433; Z = -1.126; *p* = 0.260). The prevalence ratio indicated that players without prior injuries had a protective effect (PR = 0.746; 95% CI = 0.652–0.855).

When assessing injury occurrence versus turf type, no statistically significant association was found (χ ^2^ (2) = 4.153; *p* = 0.054), though the OR suggested a marginal risk (OR = 1.981; 95% CI = 1.017–3.858; *p* = 0.042). Regarding turf type (natural versus synthetic) and treatment (conservative versus surgical), no significant association appeared (χ ^2^ (2) = 1.454; p = 0.257). The OR (3.214; 95% CI = 0.387–26.727) suggested conservative treatment was more frequent on synthetic turf, but the wide confidence interval indicated inconclusiveness.

The analysis of trauma-related injuries and treatment type revealed a non-significant trend (χ ^2^ (2) = 3.164; *p* = 0.075). Even the though OR (0.325; 95% CI = 0.09–1.168) suggested trauma could increase surgical treatment likelihood, the wide interval confirmed no statistical significance.

### Injury Incidence on Different Types of Grass: Natural and Artificial Turf

**Fig. 1:**
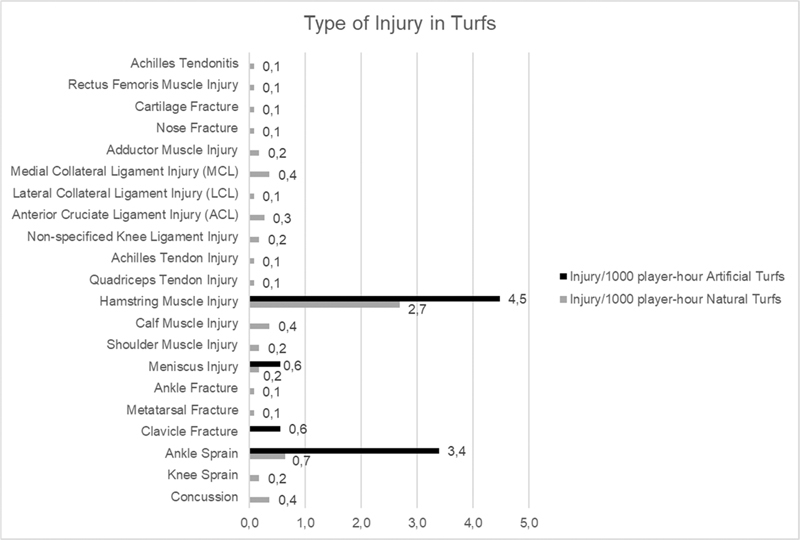
Type of Injury per 1 thousand player-hours on natural and artificial turfs.

A total of 88 injuries were recorded, corresponding to a mean of 0.23 injuries per match (95%CI: 0.17–0.28), 71 on natural turf throughout 326 matches, and 17 on artificial turf throughout 54 matches. When adjusted for exposure, the hamstring-injury incidence was of 4.5 per 1 thousand player-hours on artificial turf, and of 2.7 per 1 thousand player-hours on natural turf ( Fig. 1 ). When normalized by exposure, the incidence was of 6.6 injuries per 1 thousand player-hours on natural turf, and 9.5 injuries per 1 thousand player-hours on artificial turf. The IRR comparing artificial to natural turf was of 1.44 (95%CI: 0.85–2.45; *p* = 0.18), although the 95%CI included the unit. The incidence of ankle sprains was of 3.4 per 1 thousand player-hours on artificial turf compared with 0.7 per 1 thousand player-hours on natural turf ( Fig. 1 ). Based on the publicly-available information regarding previously-reported injuries in the same anatomical region, the difference in ankle-sprain incidence was attenuated.

### Home Team Advantage: Artificial and Natural Turf

**Fig. 2:**
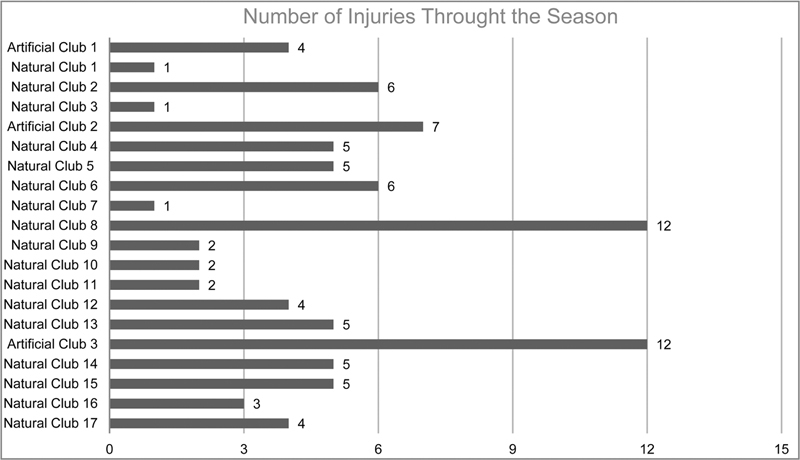
Number of injuries throughout the season.

Two teams—one whose stadium used artificial turf and one whose stadium used natural turf—recorded identical injury counts (12 each) during the season, despite differing exposure profiles ( Fig. 2 ).

### Injury Incidence Among Artificial Turf Types

**Fig. 3:**
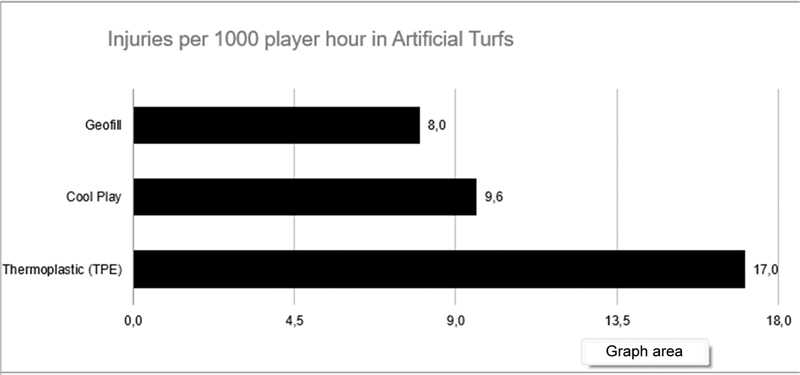
Injuries per 1 thousand player-hours according to the type of artificial and natural turf.

**Fig. 4:**
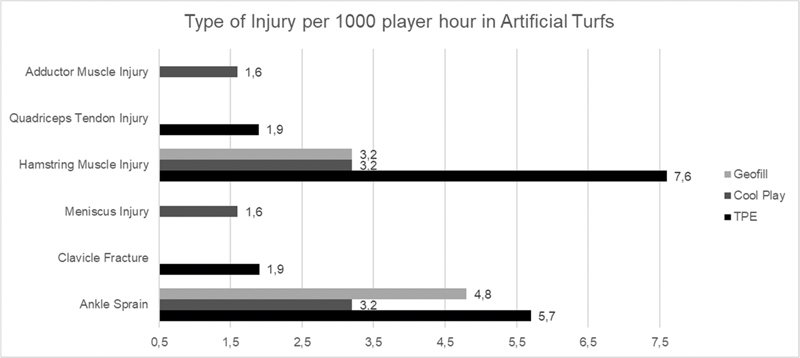
Type of injury per 1 thousand player-hour on artificials turf.

In total, 16 matches were played on TPE turf, resulting in an incidence of 17 injuries per 1 thousand player-hours. Coolplay turf hosted 19 matches, with an incidence of 9.6 injuries per 1 thousand player-hours, and Geofill turf also hosted 19 matches, with an incidence of 8.0 injuries per 1 thousand player-hours ( Fig. 3 ). After adjustment for previous injuries and direct trauma mechanisms, the incidence decreased to 11.4 injuries per 1 thousand player-hours on TPE, 9.6 injuries per 1 thousand player-hours on Coolplay, and 6.4 injuries per 1 thousand player-hours on Geofill. The TPE turf showed higher observed rates for hamstring muscle injuries (7.6) and ankle sprains (5.7). On Coolplay surfaces, hamstring injuries and ankle sprains were both recorded at 3.2 per 1 thousand player-hours, while adductor muscle injuries and meniscus injuries appeared at 1.6 per 1 thousand player-hours. Geofill turf presented lower overall values, with ankle sprains at 4.8 per 1 thousand player-hours and hamstring muscle injuries at 3.2 per 1 thousand player-hours, whereas other injury types remained below 2.0 per 1 thousand player-hours ( Fig. 4 ).

### Injury Incidence Among Natural Turf Types

**Fig. 5:**
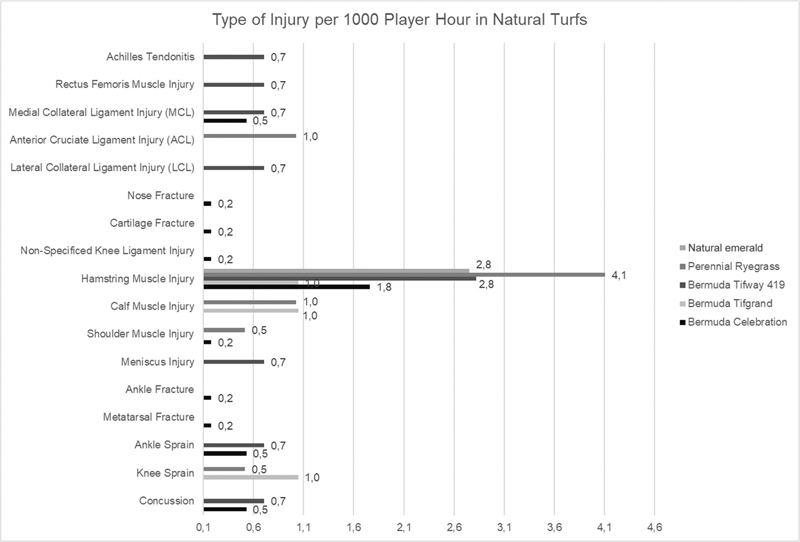
Type of injury per 1 thousand player-hour on naturals turf.

Among natural turf types, perennial ryegrass showed 7.6 injuries per 1 thousand player-hours across 60 matches. Celebration Bermuda presented 4.6 injuries per 1 thousand player-hours across 172 matches, while Tifway 419 presented 7.0 injuries per 1 thousand player-hours across 43 matches. Injury incidence rates were also observed on TifGrand (3.1 injuries per 1 thousand player-hours across 29 matches) and *esmeralda natural* (2.8 injuries per 1 thousand player-hours across 22 matches) ( Fig. 3 ). After adjustment for previous injuries and trauma mechanisms, the overall injury incidence decreased. When injuries on natural turf are expressed per 1 thousand player-hours, posterior thigh injuries appear with the highest observed values, particularly on perennial ryegrass (4.1), followed by *esmeralda natural* grass (2.8), and Celebration Bermuda (2.0). Calf injuries ranged from 1.0 per 1 thousand player-hours on perennial ryegrass to values below 1.0 on other natural surfaces. Knee ligament injuries were recorded at low absolute values, with anterior cruciate ligament injuries reaching 1.0 per 1 thousand player-hours on perennial ryegrass, while medial and lateral collateral ligament injuries generally remained around 0.7 or lower across grass types. Other injury types, including meniscal injuries, ankle sprains, fractures, shoulder injuries, and concussions, were observed at low frequencies, typically between 0.2 and 0.7 injuries per 1 thousand player-hours, with small variations among the different natural grass surfaces ( Fig. 5 ).

### Match Time and Weather Conditions

**Fig. 6:**
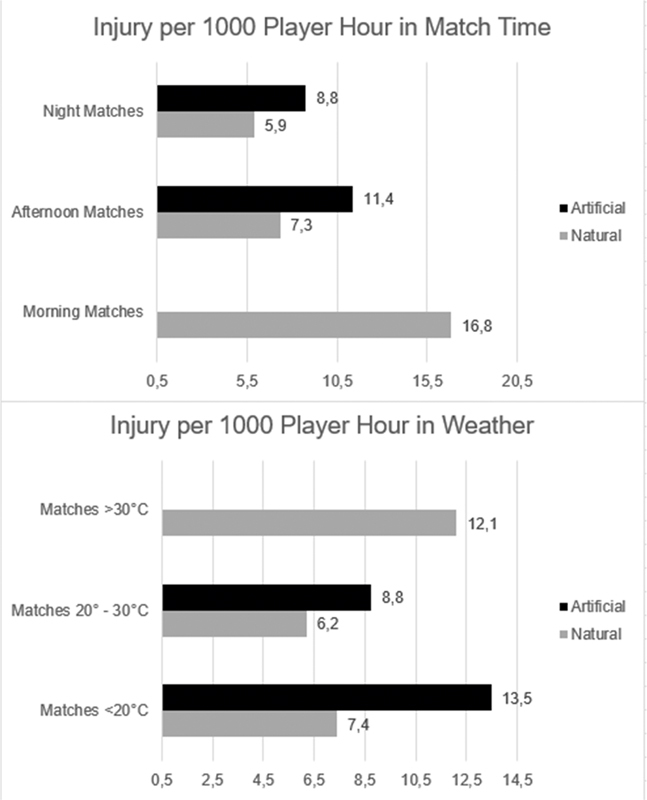
Injury per 1 thousand player hour according to match time and weather conditions.

When injury incidence was described according to match time, different distributions were observed across turf types. In morning matches, injuries were recorded only on natural turf, corresponding to an incidence of 16.8 injuries per 1 thousand player-hours. During afternoon matches, the incidence on artificial turf was of 11.4 injuries per 1 thousand player-hours, whereas natural turf presented 7.3 injuries per 1 thousand player-hours. In night matches, the incidence reached 8.8 injuries per 1 thousand player-hours on artificial turf and 5.9 injuries per 1 thousand player-hours on natural turf. Regarding weather conditions, injury incidence varied across temperature ranges and playing surfaces. In matches played under 20 °C, incidence values of 13.5 injuries per 1 thousand player-hours on artificial turf and 7.4 injuries per 1 thousand player-hours on natural turf were observed. Between 20 °C and 30 °C, the incidence corresponded to 8.8 injuries per 1 thousand player-hours on artificial turf and 6.2 injuries per 1 thousand player-hours on natural turf. In matches played at temperatures above 30 °C, injuries were only recorded on natural turf, with an incidence of 12.1 injuries per 1 thousand player-hours, as no matches on artificial turf occurred under these conditions ( Fig. 6 ).

### Type of Treatment and Recovery Time

Of the 71 injuries sustained on natural turf throughout 326 matches, 12 corresponded to a surgical treatment incidence of 1.1 surgeries per 1 thousand player-hours, while 59 injuries resulted in a conservative treatment incidence of 5.5 per 1 thousand player-hours. On artificial turf, 17 injuries occurred over 54 matches, with a surgical treatment incidence of 0.6 surgeries per 1 thousand player-hours. The overall injury-related recovery time did not differ significantly between surfaces (mean difference = 0.43 weeks; *p* > 0.05), with an average difference of 0.43 weeks (approximately 4 days), being slightly longer for lesions on natural turf.

Overall, the analysis revealed consistent patterns. Lower-limb injuries predominated regardless of surface type, with hamstring strains and ankle sprains representing the most frequently observed injury categories. Artificial turf tended to show higher injury incidence per match, particularly for muscle and ankle-related injuries, although differences between surfaces were generally small after exposure to normalization. Among turf subtypes, greater variability was observed on specific artificial systems and on perennial ryegrass among natural surfaces. Temporal and environmental analyses indicated higher injury incidence during afternoon and night matches and under cooler temperatures on artificial turf, whereas higher temperatures were associated with increased injury incidence on natural grass. Collectively, the data highlight surface-specific trends influenced by exposure, temperature, and match conditions, without demonstrating large or consistent disparities between turf types.

## Discussion

The current study provides an epidemiological analysis of injury rates in Brazilian men's professional football and demonstrates that, after exposure normalization, differences between artificial and natural turf were generally small, despite the presence of some surface-specific patterns. In the 2024 Campeonato Brasileiro Série A, the injury incidence per 1 thousand player-hours was numerically higher on artificial turf compared with natural grass (9.5 versus 6.6 respectively), without sufficient statistical evidence to support a causal relationship. The observed association may reflect surface-related characteristics; however, the retrospective design of the study does not enable mechanistic or causal inferences.

In contrast, Kuitunen et al. 8 reported a 21% lower injury incidence on artificial turf among professional players. These findings, however, may not be directly comparable to the present results due to differences in competitive context, injury surveillance systems, surface standardization, and sport-specific biomechanical demands.

Among the 3 teams that played home matches on artificial turf, only 1 demonstrated an injury incidence above the league average, reaching 17 injuries per 1 thousand player-hours, with 7.6 hamstring injuries and 5.7 ankle sprains per 1 thousand player-hours. This team played on TPE turf, the only surface of this type among the analyzed stadiums, whereas Coolplay and Geofill systems showed similar incidence rates. These findings suggest that variability within artificial turf systems may be influenced by multiple contextual factors, including surface characteristics, maintenance quality, training load, players' clinical history, playing style, and environmental conditions during matches. 8 13 15

Consistent with the results, artificial turf—particularly TPE—showed a higher observed incidence of specific injury types, notably thigh muscle injuries and ankle sprains. Thigh muscle injuries were predominantly hamstring strains, with an incidence of 4.5 injuries per 1 thousand player-hours on artificial turf compared with 2.7 on natural grass. Ankle injuries, particularly sprains, showed the greatest discrepancy between surfaces, with an incidence of 3.4 per 1 thousand player-hours on artificial turf versus 0.7 on natural turf, in agreement with previous literature. 4 6 23 However, this difference was attenuated after excluding injuries associated with direct trauma and those with reported prior injury in the same anatomical region.

The most common mechanisms associated with ankle injuries include inversion, eversion, and rotational stress, potentially influenced by the interaction between footwear and playing surface. In contrast, injuries involving other regions, such as the hip and knee, did not demonstrate relevant differences in incidence in the comparison between natural and artificial turf. It should also be noted that the substantially-lower number of matches played on artificial turf (54 versus 326) may have limited the detection of less frequent injuries and influenced injury distribution patterns. 2 6 23 24

Additional factors such as regional climate, non-sporting use of stadiums, maintenance frequency and quality, and short recovery intervals between matches may significantly affect pitch conditions and, consequently, injury risk. More robust comparisons between surfaces would require equivalent maintenance standards and playing conditions, which are difficult to achieve in real-world professional football settings. 23

Regarding match timing, most injuries occurred during afternoon and night matches, particularly on artificial turf. The small number of morning matches—with only five injuries recorded on natural turf and none on artificial turf—limits direct comparisons across time periods. Morning matches showed higher injury incidence on natural turf, whereas afternoon and night matches demonstrated numerically-higher incidence on artificial surfaces, suggesting that injury risk may vary according to surface type and match timing.

The temperature of the environment also influenced injury incidence. In matches played below 20 °C, injury incidence was higher on artificial turf (13.5 versus 7.4 injuries per 1 thousand player-hours). Conversely, higher temperatures were associated with increased injury incidence on natural grass. These findings differ from those of studies on American college football, in which lower temperatures were associated with reduced injury rates on artificial turf, indicating that the relationship between temperature, surface type, and injury risk is sport- and context-dependent. 15

With respect to treatment, injuries sustained on artificial turf were more frequently managed conservatively, whereas injuries on natural grass were more often associated with surgical intervention. However, treatment modality alone does not necessarily reflect injury severity, and these findings should be interpreted cautiously, given the descriptive nature of the data.

Finally, individual athlete characteristics, together with surface quality and maintenance, remain key determinants of injury risk and recovery. Accordingly, the findings of the present study should be interpreted as descriptive and exploratory, providing an overview of injury occurrence and distribution across turf types, without supporting definitive conclusions regarding causality or injury prevalence related to specific playing surfaces. 15

### Limitations

An important limitation to the current study relates to the method used for injury data collection, which relied exclusively on secondary, publicly-available sources, including official club communications and media reports. Although these sources enable a broad overview of injury occurrence throughout the season, the absence of a standardized injury-surveillance system and direct clinical assessment may compromise the accuracy of information regarding injury diagnosis, severity, mechanism, and therapeutic management. Consequently, underreporting—particularly of minor injuries—misclassification, and heterogeneity in injury definitions across sources cannot be ruled out and represent inherent limitations of the study design.

Although international the injury-surveillance systems proposed by FIFA, UEFA, and the IOC provide standardized methodological guidance, their full application was not feasible due to the exclusive reliance on secondary, publicly-available data sources.

In addition, return-to-play information was unavailable for approximately 38% of the reported injuries, limiting the robustness of analyses related to recovery time and injury severity. This limitation further reflects the dependence on the completeness and accuracy of publicly-reported data.

Another relevant limitation concerns exposure imbalance. Most matches were played on natural grass, as only 3 of the 20 stadiums in the league used artificial turf. As a result, 54 matches were played on synthetic surfaces, compared with more than 300 on natural grass, which may have reduced the statistical power to detect differences, particularly for less frequent injury types.

Moreover, the study population was restricted to male professional football players competing in the Campeonato Brasileiro Série A. Therefore, the findings may not be generalizable to female players, youth categories, amateur levels, or to athletes of other sports played on artificial turf.

The lack of control over several potential confounding factors represents an additional limitation. Variables such as training load, playing style, footwear, detailed injury history, pitch wear throughout the season, maintenance practices, and structural or climatic differences between stadiums could not be systematically controlled and may have influenced the observed injury patterns.

Finally, several parameters explored in the present study—such as home advantage, match time, environment temperature, and analyses stratified by specific artificial turf systems—remain sparsely investigated in the existing literature. This scarcity limits direct comparisons and deeper contextual interpretation of the findings. Future prospective studies using standardized injury surveillance systems and comprehensive control of confounding variables are warranted to confirm and expand upon these observations.

## Conclusion

In the context of the Campeonato Brasileiro Série A, a numerically-higher incidence of musculoskeletal injuries was observed on artificial turf compared with natural turf, with hamstring strains more common on natural surfaces and ankle sprains more frequent on artificial turf. Overall, the differences in injury occurrence between turf types were minimal.

Injury frequency was also associated with climatic conditions, with higher occurrence on artificial turf at lower temperatures and on natural turf at higher temperatures. Injuries requiring surgical treatment were more frequently reported on natural turf, although recovery-time differences in the comparison between surfaces were small.

These findings represent observational associations and should be interpreted with caution due to methodological limitations, including reliance on secondary data and imbalanced exposure, and they do not enable definitive conclusions regarding injury prevalence by turf type. Prospective studies with standardized injury surveillance are warranted.
